# Exploring complement-dependent cytotoxicity by rituximab isotypes in 2D and 3D-cultured B-cell lymphoma

**DOI:** 10.1186/s12885-022-09772-1

**Published:** 2022-06-20

**Authors:** Sandra Lara, Juliane Heilig, Alexander Virtanen, Sandra Kleinau

**Affiliations:** 1 Department of Cell and Molecular Biology, Uppsala University, Uppsala, Sweden; 2 Department of Biomedicine, University of Basel, Basel, Switzerland

**Keywords:** Rituximab, Isotypes, B-cell lymphoma, 3-dimensional, Spheroids, Complement-dependent cytotoxicity, CD20, CD59

## Abstract

**Background:**

The therapeutic IgG1 anti-CD20 antibody, rituximab (RTX), has greatly improved prognosis of many B-cell malignancies. Despite its success, resistance has been reported and detailed knowledge of RTX mechanisms are lacking. Complement-dependent cytotoxicity (CDC) is one important mode of action of RTX. The aim of this study was to systematically evaluate factors influencing complement-mediated tumor cell killing by RTX.

**Methods:**

Different RTX isotypes, IgG1, IgG3, IgA1 and IgA2 were evaluated and administered on four human CD20^+^ B-cell lymphoma cell lines, displaying diverse expression of CD20 and complement-regulatory protein CD59. Complement activation was assessed on lymphoma cells grown in 2 and 3-dimensional (3D) culture systems by trypan blue exclusion. CDC in 3D spheroids was additionally analyzed by Annexin V and propidium iodide staining by flow cytometry, and confocal imaging. Anti-CD59 antibody was used to evaluate influence of CD59 in RTX-mediated CDC responses. Statistical differences were determined by one-way ANOVA and Tukey post hoc test.

**Results:**

We found that 3 out of 4 lymphomas were sensitive to RTX-mediated CDC when cultured in 2D, while 2 out of 4 when grown in 3D. RTX-IgG3 had the greatest CDC potential, followed by clinical standard RTX-IgG1 and RTX-IgA2, whereas RTX-IgA1 displayed no complement activation. Although the pattern of different RTX isotypes to induce CDC were similar in the sensitive lymphomas, the degree of cell killing differed. A greater CDC activity was seen in lymphoma cells with a higher CD20/CD59 expression ratio. These lymphomas were also sensitive to RTX when grown in 3D spheroids, although the CDC activity was substantially reduced compared to 2D cultures. Analysis of RTX-treated spheroids demonstrated apoptosis and necrosis essentially in the outer cell-layers. Neutralization of CD59 overcame resistance to RTX-mediated CDC in 2D-cultured lymphoma cells, but not in spheroids.

**Conclusions:**

The results demonstrate that CDC outcome in CD20^+^ B-cell lymphoma is synergistically influenced by choice of RTX isotype, antigen density, tumor structure, and degree of CD59 expression. Assessment of tumor signatures, such as CD20/CD59 ratio, can be advantageous to predict CDC efficiency of RTX in vivo and may help to develop rational mAbs to raise response rates in patients.

**Supplementary Information:**

The online version contains supplementary material available at 10.1186/s12885-022-09772-1.

## Background

Monoclonal antibodies (mAbs) represent a fast-growing class of biological therapeutics that have shown to be one of the most successful strategies for the treatment of hematological malignancies and solid tumors [1–3]. The therapeutic effect of anti-cancer mAbs stems from their capacity to opsonize targeted cancer cells and either induce direct cell killing (apoptosis) or through Fc-dependent immune effector mechanisms, such as antibody-dependent cell-mediated cytotoxicity (ADCC), antibody-dependent cell-mediated phagocytosis (ADCP), and complement-dependent cytotoxicity (CDC) [4–6].

CDC is an important effector mechanism for antibody-mediated clearance of target cells. Briefly, upon binding to target cells, the antibody’s Fc domain recruits complement component C1. The C1 complex, consisting of the antibody recognition protein C1q and serine proteases, C1r and C1s (C1r_2_s_2_), initiates a proteolytic cascade resulting in the formation of the membrane attack complex (MAC) into the target cell membrane, generating lytic pores and cell death [7, 8]. In particular, surface-bound IgG molecules that organize into hexamers, forming a strong platform for C1q docking, have been reported to be efficient in inducing complement activation [9]. This emphasizes the importance of antibody clustering on the target surface to facilitate avid C1q binding and efficient activation.

To prevent normal host cell damage by incidentally activated autologous complement, host cells express membrane-bound complement regulatory proteins such as CD46, CD55 and CD59. CD59 inhibits MAC formation and is very effective in protecting cells from complement-mediated lysis [10]. Tumor cells themselves can express variable degrees of CD59 to evade destruction by complement, and the resistance to CDC can even be much higher than that of normal cells [11–13].

The role of CDC in the killing of malignant cells has best been described in the collection of therapeutic anti-CD20 mAbs, including rituximab (RTX), a murine-human chimeric IgG1. RTX has demonstrated in vitro efficacy promoting CDC in CD20^+^ B-cell lymphoma cell lines, while the CDC activity in vivo is less established [12, 14, 15]. Though, a strong argument that complement has a role in vivo is the observation that the clinical response to RTX in B-cell cancer is improved after supplementation with fresh-frozen plasma [16].

Although many of the currently approved mAb therapies are of the IgG1 isotype, other human isotypes may also be efficient in tumor cell killing as they can engage complement proteins and Fc receptors differently [17]. In a previous study we demonstrated that RTX-IgG3 possesses superior effector functions over RTX-IgG1 in CD20-positive B-cell lymphoma and that lymphoma cells grown in 3D cultures (spheroids) are more resistant to treatment than conventional 2D monolayer cultures [18]. Multicellular spheroids have an advantage in immunotherapy research as they have a characteristic tumor-like structure with a necrotic core, an inner layer of quiescent cells, and a layer of proliferating cells, which have greater power to predict clinical efficacies [19, 20].

To systematically define features that can affect CDC outcome in CD20+ B-cell lymphoma we evaluated clinical standard RTX-IgG1 in comparison with other isotypes such as IgG3, IgA1, and IgA2, which have displayed promising activity in preclinical models. The antibodies were administered to 2D and 3D cultures of four different human B-cell lymphomas to enable influence of differential tumor antigen expression and tumor structure in the CDC activity.

Here we show for the first time that tumor CD20/CD59 expression ratio, tumor architecture and choice of isotype synergistically influence RTX-mediated CDC activity in B-cell lymphoma.

## Material and methods

### Antibodies

RTX isotypes; human IgG1, IgG3, IgA1, and IgA2 anti-human CD20 mAbs, were purchased from InvivoGen, Toulouse, France. Human isotype controls; IgG1, IgG3, and IgA1 mAbs were from Bio-Rad, California, USA. PE-conjugated mouse IgG2a anti-human CD20 (clone LT20) was from EuroBioScience, Friesoythe, Germany. PE-conjugated and unconjugated mouse IgG2a anti-human CD59 (clone p282) were from BioLegend, San Diego, CA, USA. For CD59 blocking experiments 25-50 μg/mL of unconjugated mouse IgG2a anti-human CD59 was used.

### Cell lines and culture conditions

Raji, Daudi, and BJAB cells (all human Burkitt’s B-cell lymphoma), authenticated by STR-profiling (Microsynth AG, Switzerland), were cultured in RPMI (Gibco, Waltham, MA, USA) supplemented with 1% penicillin and streptomycin (Sigma-Aldrich). GRANTA-519 cells (human mantle B-cell lymphoma) were purchased from DSMZ, Braunschweig, Germany and cultured in Dulbecco’s Modified Eagle’s Medium (DMEM) (Sigma-Aldrich, St. Louise, MO, USA) supplemented with 1% penicillin and streptomycin. All cell cultures were supplemented with 10% heat-inactivated fetal bovine serum (Gibco, Waltham, MA, USA) and cultured at 37 °C in a humidified chamber under 5% CO_2_ following manufacturer’s recommendations. Cells were routinely screened for mycoplasma contamination using MycoAlert plus detection kit (Lonza, Basel, Switzerland).

### Spheroids

B-cell lymphomas grown in 3D (spheroids) were obtained by culturing lymphoma cells following the procedure previously described [18, 20]. In brief, 0.15 g of agarose were added to 10 mL of a solution containing 10% of DMEM and 90% of PBS, and subsequently autoclaved. Next, each well of a 96-well round-bottomed plate (Sarstedt, Newton, NC, USA) was coated with 50 μL of the agarose solution under aseptic conditions. To obtain Daudi and GRANTA-519 spheroids, 10,000 cells/well were seeded, while BJAB and Raji spheroids were obtained by seeding 5000 cells/well in 200 μL of complete culture media. After cell seeding, plates were centrifuged for 6 min at room temperature at 200 rcf. Subsequently, plates were incubated at 37 °C in a humidified chamber of 5% CO_2._ The size and morphology of the spheroids were defined using an inverted microscope (Leica DMi1, Leica Microsystems, Wetzlar, Germany). Spheroids obtained after 2 days of culture were subsequently used for further experiments.

### CDC

The CDC experiments were conducted with human plasma as a complement source, isolated from freshly drawn blood in heparin-coated tubes of anonymous healthy blood donors. As anti-coagulants such as heparin may interfere with complement [21] the isolated plasma was tested and confirmed to promote antibody-mediated CDC, while heat-inactivated plasma did not.

#### CDC in monolayers

Following a previously described protocol [18], lymphoma B-cells were seeded in a 96-well round-bottomed plate using a cell density of 50,000 cells/well in 50 μL of complete cell culture media. The cells were then incubated with 5 μg/mL of the different RTX isotype variants or human isotype control antibodies for 30 min at 37 °C. Subsequently, 25% of human plasma (or inactivated after incubation at 56 °C for 30 min) was added and incubated for further 30 min at 37 °C. Viable cells were then determined by counting the cells on a Neubauer hemocytometer using the trypan blue exclusion method (% viable cells = 100 x (live cells)/(live cells + dead cells).

#### CDC in spheroids

 Spheroids were incubated with 10 μg/mL of different RTX isotypes or human isotype control antibodies for 30 min at 37 °C, as previously described [18]. Next, 25% human plasma, or heat-inactivated human plasma, was added and incubated for further 24 h at 37 °C. After incubation, the cell viability of each disaggregated spheroid was determined by cell counting on a Neubauer hemocytometer using the trypan blue exclusion method. In parallel, necrotic and apoptotic cells were determined by propidium iodide (PI) (Sigma-Aldrich) and Annexin V FITC (BioLegend) staining at 5 μg/mL for 15 min on ice or 0.45 μg/mL for 15 min at room temperature protected from light respectively using flow cytometer (MACSQuant VYB). Data were analyzed using FlowJo software (version 10.6.2). Gating strategy can be seen in supplementary information figure 1.

### Imaging of 3D B-cell lymphomas

Raji spheroids treated with RTX and 25% human plasma were washed with PBS and stained with PI (Sigma-Aldrich) or Annexin V FITC (BioLegend) together with Hoechst 33342 (Invitrogen) for 15 min at room temperature protected from light. The spheroids were thereafter washed with PBS, fixed with 2% PFA for 10 min at 37 °C, and carefully put on microscope slides (Superfrost Plus, ThermoScientific). Raji spheroids were imaged in glycerol at room temperature using an LSM 710 Elyra S.1, AxioObserver confocal microscope equipped with 405, 488, and 561 nm lasers and Plan-Apochromat 10x/0.3 DIC M27. Images were acquired using Zen (Black edition) software. All images were analyzed using the open-source Java application ImageJ (https://imagej.nih.gov/ij/). The profile of Annexin V FITC and PI mean fluorescence intensity of CDC in the RTX-treated spheroids was measured using a macro. Briefly, this macro automatically thresholds the image, selects the largest threshold object and produces the mean intensity from the spheroid periphery towards the center from Z projected (maximum intensity) z-stack images.

### Statistics

CDC data is presented as mean ± SEM of at least 3 independent experiments. Statistical differences between groups were determined by one-way ANOVA with Tukey post hoc test for multiple comparisons using GraphPad Prism 8 software (version 8.4.2). Asterisks were used to indicate statistical significance: *p < 0.05, **p < 0.01, ***p < 0.001, ****P < 0.0001.

## Results

### Efficacy of RTX to induce CDC is dependent on antibody isotype and CD20/CD59 expression ratio in B-cell lymphoma cells

**Fig. 1:**
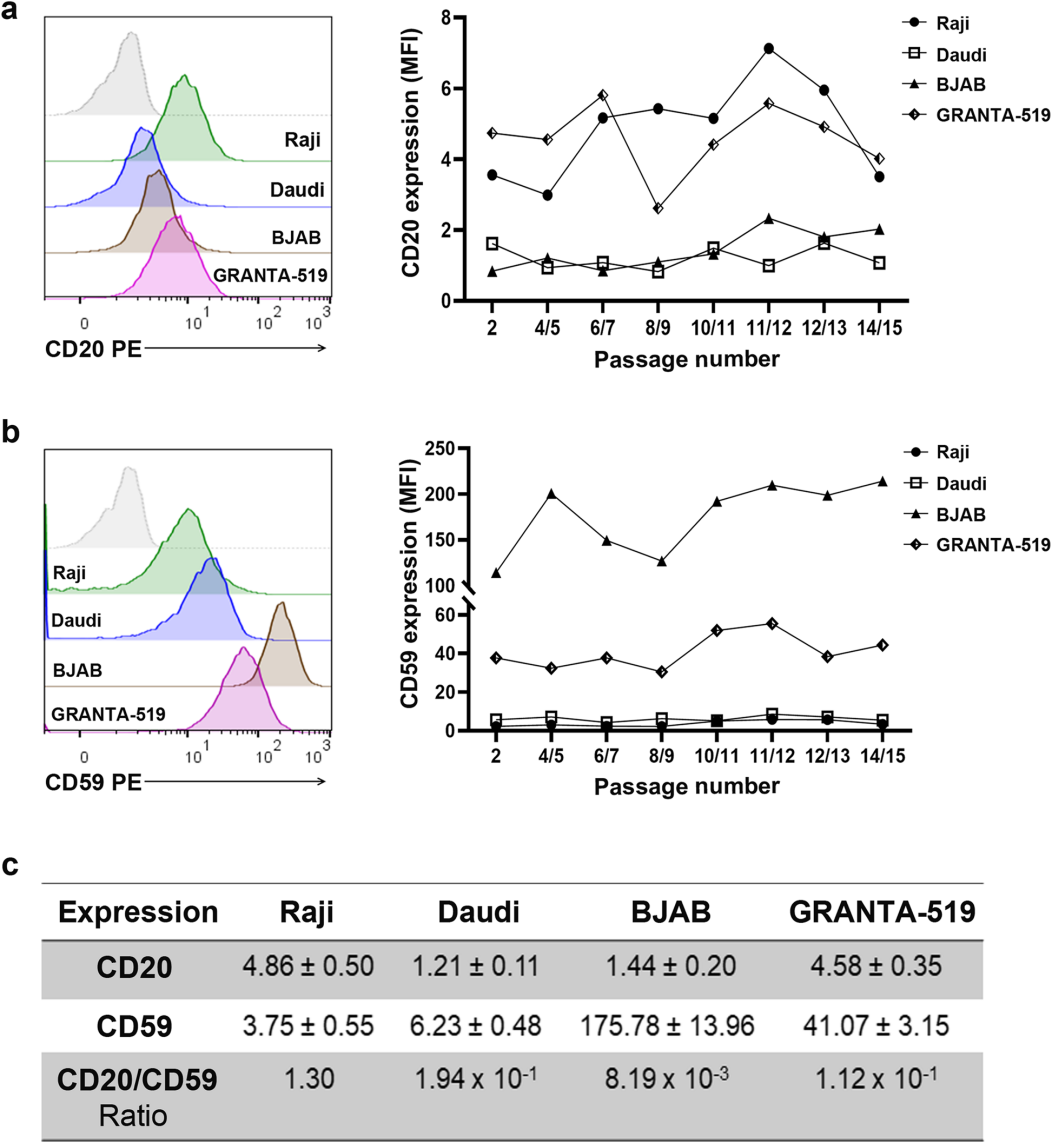
CD20 and CD59 expression in four B-cell lymphoma cell lines. Raji, Daudi, BJAB, and GRANTA-519 cells were cultured in 2D monolayers and stained with anti-CD20 or anti-CD59 antibody, and analyzed by flow cytometry. Representative histograms of cells stained with phycoerythrin (PE)-labeled anti-CD20 (**a**) and anti-CD59 (**b**) antibody at passage number 11/12. Grey histogram represents isotype control. Graph represents the median fluorescence intensity (MFI) of CD20 (**a**) and CD59 (**b**) on passage number 2-15 with corresponding isotype control subtracted. **c** Mean of MFI of passage number 2-15 with corresponding isotype control subtracted ± SEM of 8 independent experiments, and ratio of mean MFI of CD20 and CD59

Evading the immune system is a prerequisite for neoplastic progression and a hallmark of cancer [22]. To investigate how CD20^+^ B-cell lymphoma responds to RTX-mediated CDC, we first characterized four lymphoma B-cell lines, taking into account target antigen and complement regulatory protein expression. Three Burkitt’s lymphomas; Raji, Daudi, and BJAB, and one mantel cell lymphoma; GRANTA-519 were cultured in 2D and the expression levels of CD20 and CD59 were analyzed by flow cytometry. As CDC experiments were conducted on passage numbers 2-15 we determined the expression levels in each cell line over time. Overall, the CD20 expression was stable, particularly in Daudi and BJAB cells, while in Raji and GRANTA-519 cells it fluctuated slightly (Fig. 1a). The mean expression values of CD20 showed that GRANTA-519 and Raji cells had the highest CD20 expression, followed by BJAB and Daudi cells, albeit at very low levels (Fig. 1c). The CD59 expression was fairly consistent across the studied passages (Fig. 1b). BJAB cells had the highest mean CD59 expression followed by GRANTA-519, while very low CD59 was found in Daudi and Raji cells (Fig. 1c). By calculating the CD20/CD59 expression ratio in each cell line the lymphomas could be ranked (starting with the highest ratio) accordingly Raji/Daudi/GRANTA-519/BJAB (Fig. 1c).

**Fig. 2:**
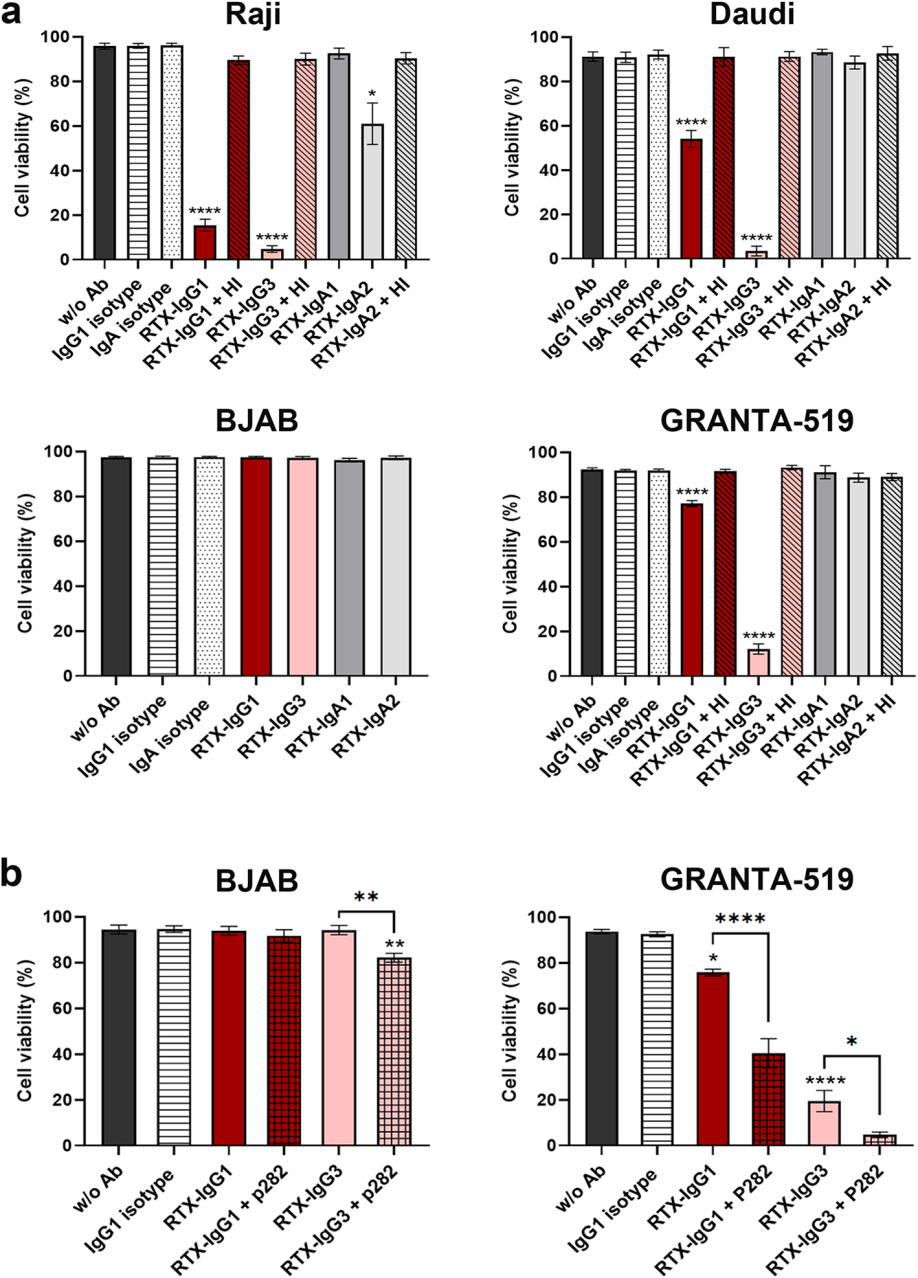
RTX-mediated CDC in 2D cultures of CD20^+^ B-cell lymphomas. Cell viability was analyzed by trypan blue exclusion in four B-cell lymphoma cell lines cultured in monolayers and supplemented with human plasma, or where indicated, with heat-inactivated (HI) plasma. **a** Percentage cell viability in Raji, Daudi, BJAB, and GRANTA-519 cells following treatment with RTX isotypes or isotype controls. **b** Percentage cell viability with or without CD59 blocking, using 50 or 25 μg/mL anti-human CD59 (p282) in BJAB and GRANTA-519 cells respectively, prior treatment with RTX isotypes. Data are presented as the mean percentage cell viability ± SEM of 3-6 independent experiments. Statistical significance was determined by one-way ANOVA with Tukey post hoc test for multiple comparisons. Asterisk represents significant difference between RTX and isotype control, or between RTX treatments (with and without CD59 blocking)

Next, we evaluated RTX-mediated CDC using the complement activating IgG1 and IgG3 subclasses, but also the less well studied IgA1 and IgA2 variants, in the four B-cell lymphoma 2D cultures, supplemented with human plasma. The complement activating function was assessed by trypan blue, which revealed high cell viability in untreated and isotype control treated cells, while reduced cell viability in three out of the four B-cell lines treated with RTX (Fig. 2a). Raji was the most susceptible cell line to CDC, followed by Daudi, and GRANTA-519 cells (Fig. 2a). BJAB cells demonstrated complete resistance to RTX-mediated CDC. IgG3 was the most potent RTX isotype inducing tumor cell killing in all CDC sensitive cell lines, while RTX-IgG1 induced efficient killing in Raji cells, but only moderately in Daudi and GRANTA-519 cells. Notably, no CDC was triggered when the culture media was supplemented with heat-inactivated human plasma, signifying the complement dependency of this process. Furthermore, by using RTX variants of IgA subclasses we observed that RTX-IgA2, but not RTX-IgA1, could reduce cell viability in Raji cells (Fig. 2a). This killing effect was not seen in other cell lines, or in Raji cell cultures supplemented with heat-inactivated plasma, signifying complement involvement.

CD59 plays an important regulatory role in CDC and a high CD59 expression may limit or hinder CDC activity [8, 23]. Thus, we next investigated if blocking of CD59 could be a strategy to overcome RTX-mediated CDC resistance in the highly CD59 expressing BJAB and GRANTA-519 cells. We used the mAb p282 (anti-human CD59) to block CD59 on BJAB and GRANTA-519 in 2D cell cultures, prior to treatment with RTX-IgG1 and RTX-IgG3. A dose of 25 μg/mL p282 could not affect the CDC resistance in BJAB cells (data not shown), however, when 50 μg/mL was used a significant killing effect with RTX-IgG3, but not RTX-IgG1, could be observed in the BJAB cells (Fig. 2b). On the other hand, when GRANTA-519 cells were blocked with 25 μg/mL of p282 both RTX-IgG1 and RTX-IgG3 could expand the killing effect of respective isotypes when used without blocking. Collectively, these results indicate that a high CD20/CD59 expression ratio in B-cell lymphoma facilitates anti-tumor immunity by RTX.

### The 3D structure of B-cell lymphoma hinders RTX-mediated CDC

**Fig. 3:**
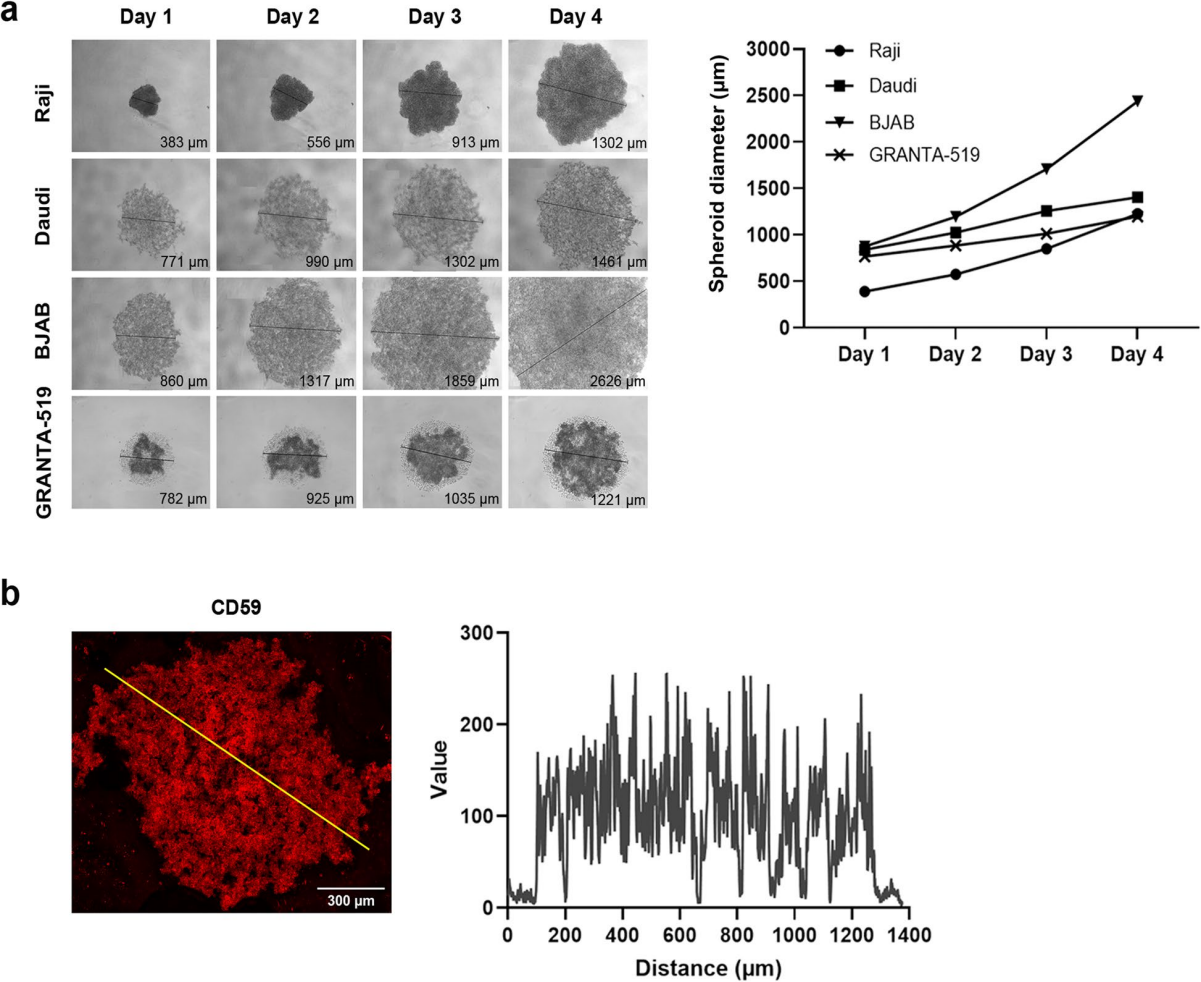
Development of 3D spheroids of CD20^+^ B-cell lymphomas and expression pattern of CD59. **a** Inverted microscopy images of 3D cultures of Raji, GRANTA-519, Daudi, and BJAB cells after 1-4 days of culture (magnification 5x) (left), and corresponding growth curves showing mean diameter of 5 spheroids per time point (right). **b** Confocal image z-stack projection of a representative GRANTA-519 spheroid stained with anti-CD59 PE antibody (red) (scale bar = 300 μm, magnification 10x). Profile of the CD59 intensity (yellow line) was examined. The X-axis represents distance along the spheroid and the Y-axis the pixel intensity

To further investigate CDC mechanism by RTX in a more clinically relevant system, 3D micro tumors were developed to better replicate tumor architecture [19]. Accordingly, the four human B-cell lymphoma cell lines were cultured on an agarose matrix, allowing cell aggregates (spheroids) to form. Within 2 days of culture spheroids of approximately 500-900 μm in diameter developed (Fig. 3a). The growth rate was similar among cell lines, except BJAB, which developed faster. Notably, the lymphoma cell lines formed spheroids of different shapes and cell densities. Daudi, BJAB, and GRANTA-519 cells formed looser spheroid structures in comparison with Raji, which formed more cell dense, compact spheroids. As CD59 affected RTX-mediated CDC in 2D cultures we wanted to understand how the CD59 antigen was exposed in spheroids. Confocal microscopy revealed that CD59 was homogeneously expressed, with a prominent appearance over the whole spheroid as demonstrated in GRANTA spheroids (Fig. 3b).

**Fig. 4:**
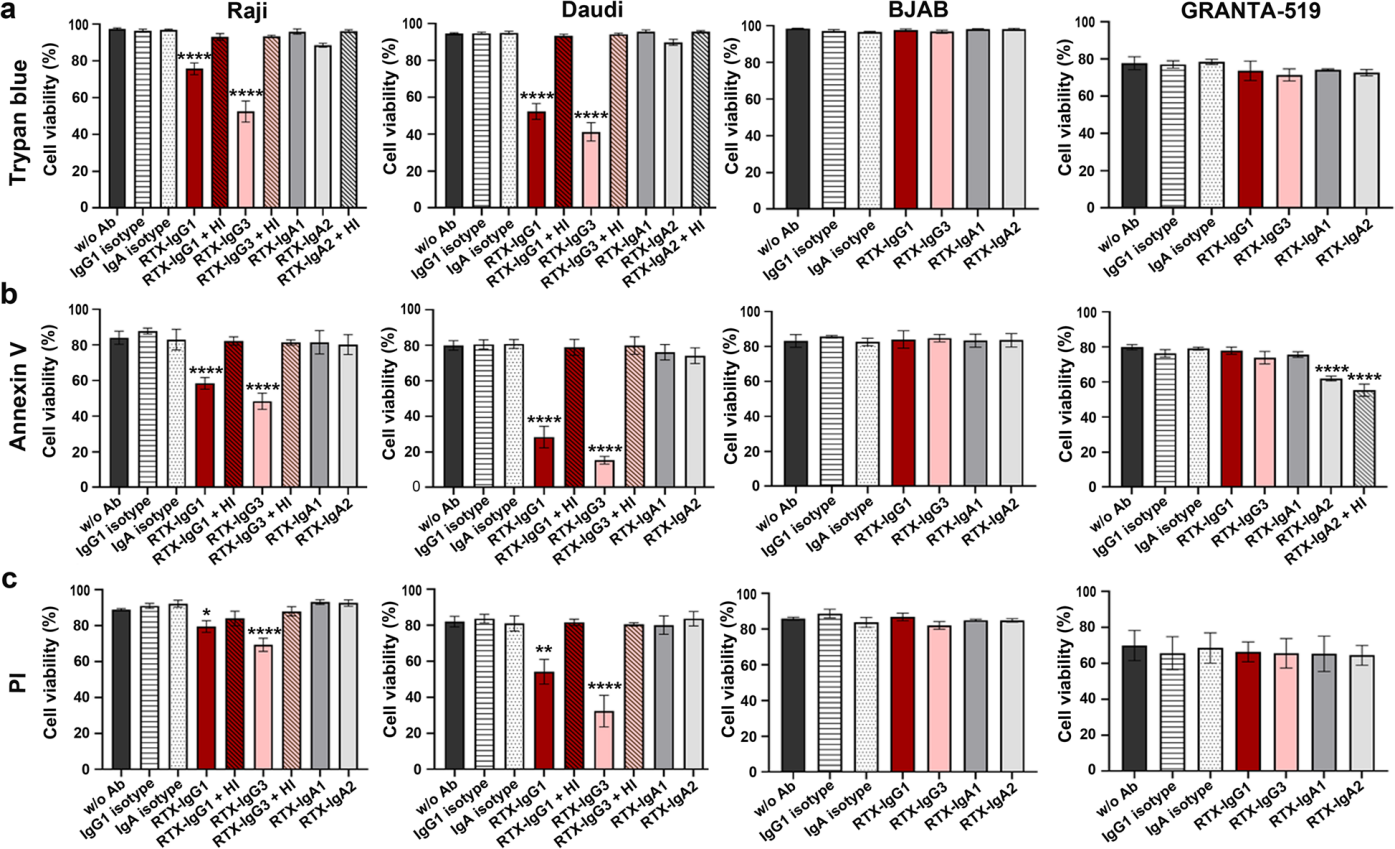
RTX-mediated killing of CD20^+^ B-cell lymphoma cultured in 3D spheroids. Percentage cell viability analyzed by **a** trypan blue **b** Annexin V or **c** propidium iodide (PI) of 3D cultured Raji, Daudi, BJAB, and GRANTA-519 cells treated with RTX isotypes or isotype controls in media supplemented with human plasma or where indicated, heat-inactivated (HI) plasma. Data are presented as the mean percentage cell viability ± SEM of 3-7 independent experiments. Statistical significance was determined by one-way ANOVA with Tukey post hoc test for multiple comparisons. Asterisk represents statistical significant difference between RTX and isotype control

Next, the efficacy of RTX isotype variants (IgG1, IgG3, IgA1, and IgA2) to mediate CDC in 3D tumor spheroids was investigated. A RTX concentration of 10 μg/mL, twice as much as in 2D, was considered most efficient in inducing cell death in 3D Raji spheroids (data not shown). RTX-mediated CDC in spheroids was thereafter evaluated by trypan blue, Annexin V, and propidium iodide (PI) staining indicative of cell viability, apoptosis, and necrosis respectively. Trypan blue exclusion revealed that RTX treatment significantly reduces cell viability in Raji and Daudi spheroids (Fig. 4a). The RTX-IgG3 variant had the best killing potential, followed by the clinical standard IgG1. RTX-IgA isotypes showed no significant effect, although a trend of decreased cell viability was seen in Raji and Daudi spheroids by RTX-IgA2 (Fig. 4a). None of the RTX isotypes tested induced cell cytotoxicity in GRANTA-519 and BJAB spheroids, nor after prior anti-CD59 treatment with p282 (data not shown). The cell cytotoxicity mediated by RTX-IgG3 and RTX-IgG1 in Raji and Daudi spheroids was not observed in 3D cultures supplemented with heat-inactivated plasma, revealing complement involvement (Fig. 4a). Flow cytometry data of Annexin V and PI staining agreed with the cell viability assessed by trypan blue, in regard to RTX-IgG3 and RTX-IgG1 being the most potent isotypes mediating apoptosis and/or necrosis in Raji and Daudi spheroids (Fig. 4b-c). Though, a significant decrease in cell viability in GRANTA-519 spheroids was observed after RTX-IgA2 treatment and Annexin V staining (Fig. 4b). This effect was not restored when supplementing the cell culture media with heat-inactivated human plasma, indicating that this cytotoxicity was likely mediated by CD20-mediated apoptosis.

When comparing the capacity of RTX isotypes to induce CDC in 3D spheroids with 2D cultures it was evident that the CDC effect was substantially reduced in spheroids (Figs. 2a and 4a). Thus, Raji spheroids treated with RTX-IgG1 or RTX-IgA2 had about 70% decreased CDC activity, while RTX-IgG3 had about 50% loss of efficacy compared with 2D cultures of Raji cells. Reduction of the complement activation capacity was also observed between 3D and 2D-cultured Daudi and GRANTA-519 cells (Figs. 2a and 4a), indicating that 3D structures of B-cell lymphoma show higher resistance to RTX-mediated CDC.

**Fig. 5:**
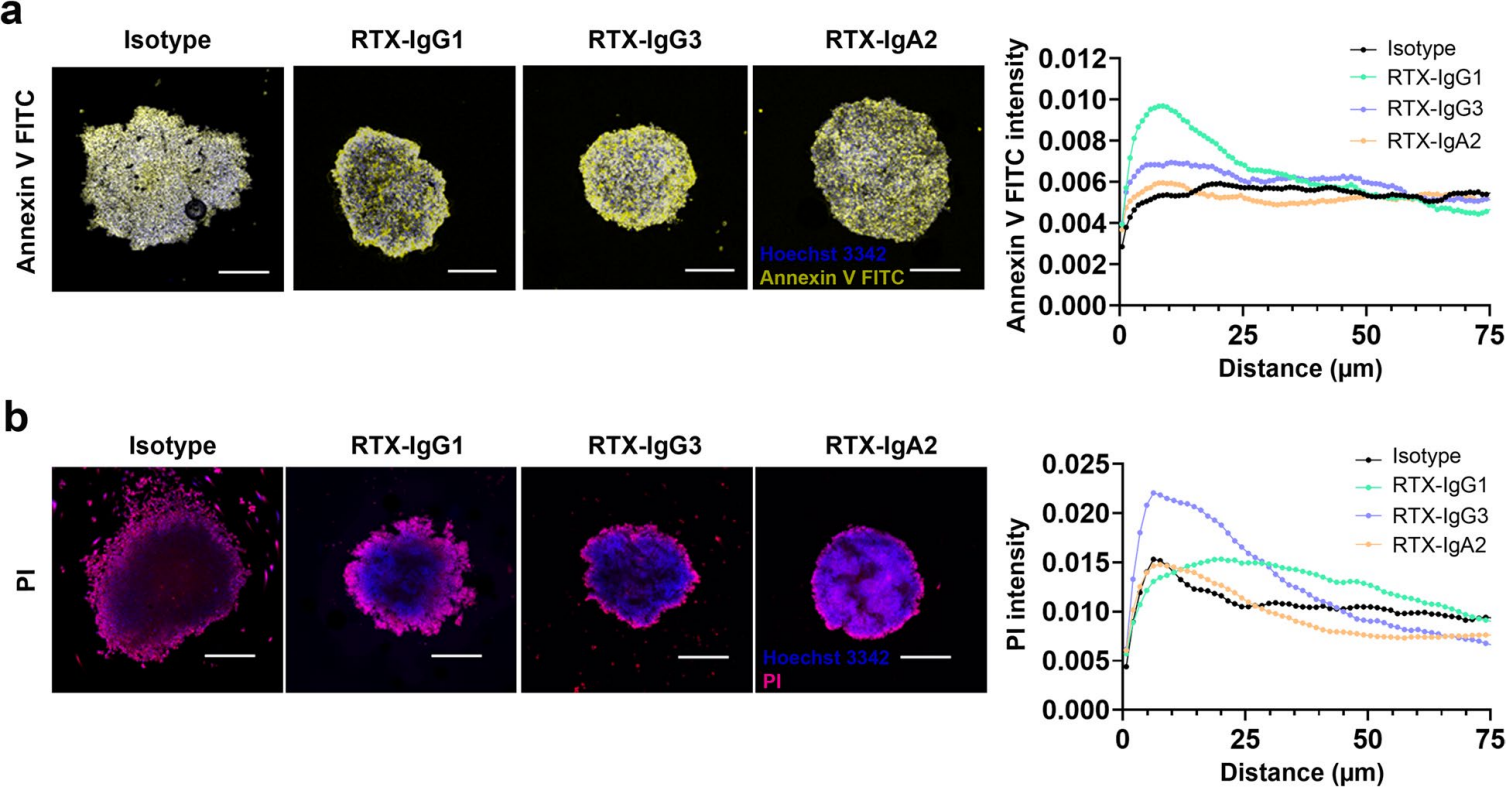
Imaging of CDC-mediated apoptosis and necrosis in Raji spheroids treated with RTX. **a** Z-projected fluorescence confocal microscopy images of Raji spheroids treated with RTX isotypes or isotype control in human plasma, and stained with Annexin V FITC (yellow) and Hoechst 33342 nuclear stain (blue). Profile of the Annexin V FITC fluorescence signal in the different RTX isotype or control-treated Raji spheroids (right panel). **b** Z-projected fluorescence confocal microscopy images of Raji spheroids treated with RTX isotypes or isotype control in human plasma, and stained with propidium iodide (PI) (pink) and Hoechst (blue). Profile of the PI fluorescence signal in the different RTX isotype or control-treated Raji spheroids (right panel). Data from one representative experiment of three independent experiments is shown. Scale bar =300 μm, magnification 10x. The X-axis in diagram represents the distance from the spheroid border and the Y-axis the pixel intensity

To understand the CDC activity by RTX in whole spheroids we used fluorescence confocal microscopy and analyzed the distance, how far into the spheroid core, the CDC process by RTX isotypes could be observed. Accordingly, cell dense Raji spheroids, treated with isotype control or RTX-IgG1, −IgG3, or -IgA2 were stained with Annexin V or PI and incubated with Hoechst nuclei stain. Confocal images revealed early apoptotic tumor cells (Annexin V positive) mostly in the outer layer of RTX-treated spheroids, although some apoptotic cells were also found towards the spheroid center (Fig. 5a). By quantitative metrics we assessed the Annexin V intensity in the spheroids showing that the RTX-isotypes induced apoptotic signals exceeding the isotype control, particularly RTX-IgG1 (Fig. 5a; right panel). The signals stretched from the periphery towards the spheroid center in RTX-IgG1 and RTX-IgG3 treated spheroids, while RTX-IgA2 induced a weak signal at the spheroid border. When PI-staining for late apoptotic/necrotic cells were analyzed in RTX-treated spheroids in the same manner, necrotic cells were abundantly seen at the outer layer of the spheroids (Fig. 5b). The profile of the PI intensity in the different spheroids showed that RTX-IgG1 and RTX-IgG3 induced higher signals than the isotype control, while RTX-IgA2 was in the range as the control (Fig. 5b; right panel). RTX-IgG3 showed substantial PI intensity in the periphery and lower activity towards the spheroid core, while RTX-IgG1 generated higher PI signal towards the spheroid core and lower in the periphery. Overall, the confocal images of RTX-mediated CDC in 3D spheroids reveal that the strongest intensity of apoptosis and necrosis is in the outer cell-layers of the spheroids (Fig. 5).

## Discussion

Tumor tissue is heterogeneous, and its composition and quality can dictate patient outcomes [24, 25]. Thus, a better understanding of tumor target cells and therapeutic mAb can lead to clinical benefits in cancer patients.

Our data reveal for the first time that susceptibility to RTX-mediated CDC in B-cell lymphoma is synergistically influenced by choice of therapeutic antibody isotype, CD20/CD59 expression ratio and tumor density. Firstly we showed that RTX, as previously demonstrated, can significantly mediate CDC in 2D B-cell lymphoma cell cultures [18, 26]. However, only three out of four B-cell lymphoma cell lines were CDC sensitive, and the degree of CDC varied in the different lymphomas, an effect also reported by others [27]. The B-cell lymphomas differed in their expression levels of the CD20 target antigen and the complement regulatory protein CD59, and lymphomas with a higher CD20/CD59 expression ratio proved to be more sensitive for RTX-mediated CDC. One explanation for this is likely the combination of CD20 with a low CD59 expression, as blocking CD59 on CD20^+^ B-cell lymphoma cells with a high CD59 expression, rendered them sensitive to RTX-mediated CDC when cultured in 2D. In line with our findings, clinical studies of B-cell lymphoma patients, treated with rituximab-cyclophosphamide, adriamycin, vincristine, and prednisone (R-CHOP), showed that high CD59 expression in tumors was associated with poor overall survival compared to patients with lower CD59 expression [28]. Thus, this indicates that CD59 expression is a good biomarker for the prognostic of RTX mAb treatment.

Moreover, by using a multi-isotype panel of RTX we uncovered that the IgG3 variant had a greater capacity of mediating CDC in the B-cell lymphomas than clinical standard IgG1. Both human IgG1 and IgG3 are potent activators of innate immunity, enabling Fc gamma receptor signaling, as well as complement activation. Between them, IgG3 has often been reported to demonstrate enhanced phagocytosis and complement activation [18, 29–31]. However, difficulties in manufacturing IgG3 due to aggregation, its shorter in vivo half-life, and rapid degradation have outweighed the potential advantages of using IgG3 as a therapeutic antibody. To circumvent these limitations, an IgG3 allotype, showing reduced aggregate formation and similar half-life as IgG1 [32], has been designed and explored for immunotherapy [33].

The structural differences among IgG subclasses relate to the hinge region, i.e., amino acid residues linking the Fab and Fc domains. IgG3 possesses a unique extended hinge compared to other IgG subclasses, which results in increased flexibility [31, 34]. The difference in flexibility and reach between Fab arms and the Fc domain affects epitope accessibility and binding valency. This allows IgG3 molecules to target low-density target antigens, less suited to ligation by other IgG types, with the potential to more effectively stimulate complement [35]. Indeed, when BJAB cells, displaying low CD20 antigen levels, were blocked with anti-CD59 we successfully triggered CDC by RTX-IgG3, but not with RTX-IgG1. While both subclasses could increase CDC activity when CD59 was blocked in GRANTA-519 cells, expressing higher CD20 levels than BJAB cells.

Furthermore, we showed that the IgA2 variant of RTX, but not IgA1, could significantly mediate CDC in Raji B-cell lymphoma cells cultured in 2D. This is in line with previous studies showing that IgA2 isotype, requiring the classical complement pathway, can induce CDC [36–38]. We further noted a significant decrease in cell viability in GRANTA-519 spheroids after RTX-IgA2 treatment. This effect was however not complement-dependent, suggesting that IgA2 can induce direct apoptosis by anti-CD20 binding. Direct apoptosis has been occasionally observed in several experimental systems by RTX [39–41].

The differential capacity to induce CDC between IgA1 and IgA2 isotypes is likely due to differences in their hinge region, being shorter in IgA2 [42, 43]. Nevertheless, despite its lack of CDC activity IgA1 has previously been shown to be an efficient variant of RTX in triggering primary monocytes for phagocytosis of 3D cultured B-cell lymphoma [18]. This fact makes IgA1 a suitable therapeutic isotype to be employed in those tumors presenting a more pro-inflammatory phenotype, where further inflammation promoted by complement activation might not be beneficial.

3D tumor spheroids represent a promising model in recapitulating the heterogeneity of human cancers, making them useful for in vitro therapeutic screening [44–46]. Indeed, 3D-cultured B-cell lymphoma demonstrated high resistance to RTX-mediated CDC, though RTX IgG1 and IgG3 could still moderately trigger complement activation in Raji and Daudi spheroids. It was not possible to overcome this resistance in highly CD59-expressing spheroids (BJAB, GRANTA-519) by CD59 blocking as evident in monolayers. This reveals that 2D cultures, despite being an initial indicator of the differential CDC activity depending on the B-cell lymphoma cell line, still lack the many features of spheroids as of adhesive and topographical forces that may modify their responses to stimuli. Truly, tumor architecture restrains RTX efficacy, observed here by apoptosis and necrosis of cells at the spheroid border, while cells toward the spheroid center are typically not affected by RTX.

## Conclusions

In this study we have identified several features that affect RTX-mediated CDC in B-cell lymphoma; CD20 antigen density, CD59 expression level, tumor structure, and choice of mAb isotype. The attributes revealed affecting CDC might also be valid for therapeutic mAbs to other tumor targets. In-depth understanding of how tumors evade immune elimination will help to further design rational therapeutic mAbs that overcome CDC resistance. Lastly, the advantages of human IgG3 in effector function motivate thoughtful reconsideration of the clinical advancement of this distinctive antibody subclass for treatment of human disease.

## Supplementary Information

**Additional file 1: Supplementary figure 1.** Gating strategy of Annexin V positive cells after RTX-mediated CDC in 3D spheroids of CD20+ B-cell lymphoma.

## Data Availability

The datasets generated and/or analyzed during the current study are available from the corresponding author on reasonable request.

## Abbreviations

ADCC: Antibody-dependent cell-mediated cytotoxicity
ADCP: Antibody-dependent cell-mediated phagocytosis
CDC: Complement-dependent cytotoxicity
mAb: Monoclonal antibody
MAC: Membrane attack complex
MFI: Median fluorescence instensity
RTX: Rituximab
2D: 2-dimensional
3D: 3-dimensional
HI: Heat-inactivated
PI: Propidium iodide
