# Supplementary material for: Exploring complement-dependent cytotoxicity by rituximab isotypes in 2D and 3D-cultured B-cell lymphoma
Source: BMC Cancer. 2022 Jun 20;22:678. doi: 10.1186/s12885-022-09772-1 (PMC9210731; doi:10.1186/s12885-022-09772-1)
Supplement: Supplementary file 1 — Additional file 1: Supplementary figure 1. Gating strategy of Annexin V positive cells after RTX-mediated CDC in 3D spheroids of CD20+ B-cell lymphoma. [file 12885_2022_9772_MOESM1_ESM.docx]

SUPPLEMENTARY INFORMATION

**Exploring complement-dependent cytotoxicity by Rituximab isotypes in 2D and 3D-cultured B-cell lymphoma**

Sandra Lara^1^, Juliane Heilig^1,2^, Alexander Virtanen^1^ and Sandra Kleinau^1^*

*Corresponding author

Email: Sandra.Kleinau@icm.uu.se


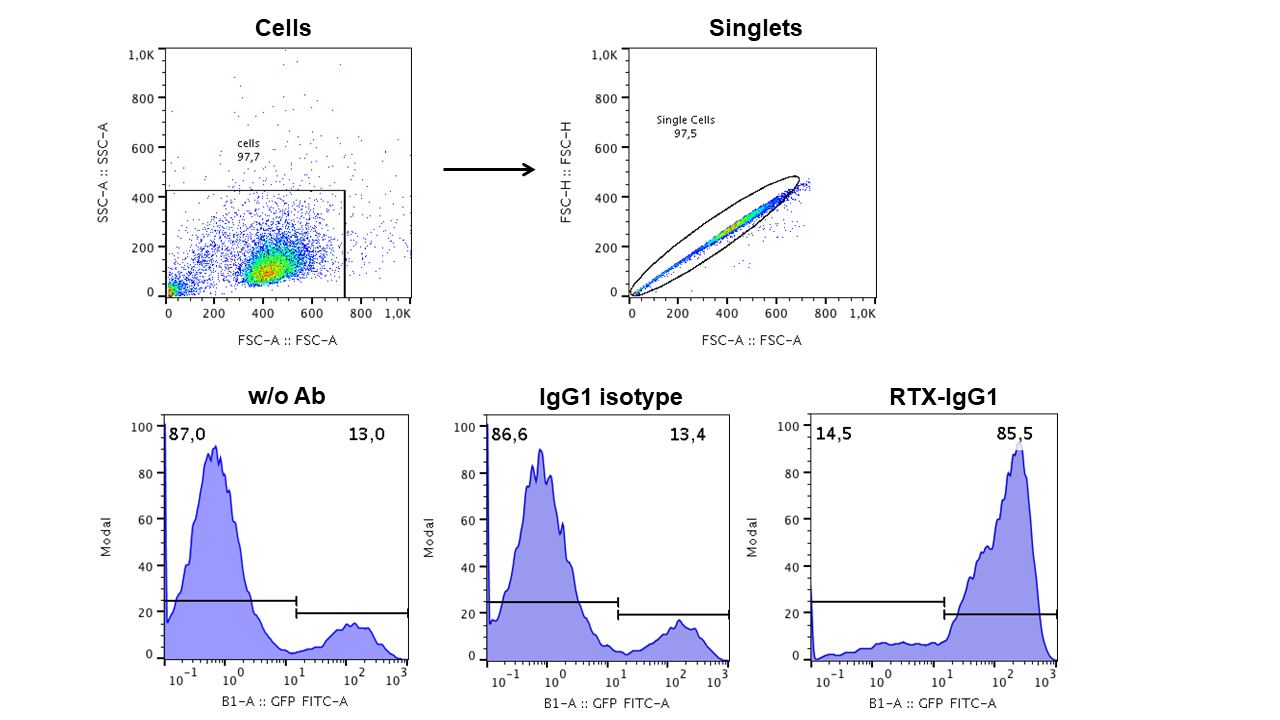


**Supplementary figure 1. Gating strategy of Annexin V positive cells after RTX-mediated CDC in 3D spheroids of CD20^+^ B-cell lymphoma.** Lymphoma cells were analysed by flow cytometry and identified on a forward scatter area (FSC-A) versus side scatter area (SSC-A) plot. Single cells were identified on a FSC-A versus FSC-H. Selected cells were further analysed for Annexin V FITC-labelled fluorescence intensity (B1-A) in a histogram. The two peaks were interpreted as positive (i.e. apoptotic cells) (right) and negative (i.e. viable cells) (left) data sets. Percentage of viable cells was quantified as the percentage of negative Annexin V FITC stained cells.
